# Mesenchymal stromal cell-derived exosome-rich fractionated secretome confers a hepatoprotective effect in liver injury

**DOI:** 10.1186/s13287-017-0752-6

**Published:** 2018-02-06

**Authors:** Apeksha Damania, Deepika Jaiman, Arun Kumar Teotia, Ashok Kumar

**Affiliations:** Department of Biological Sciences and Bioengineering, Indian Institute of Technology Kanpur, Kanpur, 208016 UP India

**Keywords:** Stromal cells, Secretome, Liver, Cryogel, Exosomes

## Abstract

**Background:**

Mesenchymal stromal cells (MSCs) are an attractive therapeutic agent in regenerative medicine. Recently, there has been a paradigm shift from differentiation of MSCs to their paracrine effects at the injury site. Several reports elucidate the role of trophic factors secreted by MSCs toward the repair of injured tissues. We hypothesize that fractionating the MSC secretome will enrich exosomes containing soluble bioactive molecules, improving its therapeutic potential for liver failure.

**Methods:**

Rat bone marrow MSCs were isolated and the conditioned media filtered, concentrated and ultracentrifuged to generate fractionated secretome. This secretome was characterized for the presence of exosomes and recovery from liver injury assessed in in-vitro liver injury models. The results were further validated in vivo.

**Results:**

Studies on in-vitro liver injury models using acetaminophen and hydrogen peroxide show better cell recovery and reduced cytotoxicity in the presence of fractionated as opposed to unfractionated secretome. Further, the cells showed reduced oxidative stress in the presence of fractionated secretome, suggesting a potential antioxidative effect. These results were further validated in vivo in liver failure models, wherein improved liver regeneration in the presence of fractionated secretome (0.819 ± 0.035) was observed as compared to unfractionated secretome (0.718 ± 0.042).

**Conclusions:**

The work presented is a proof of concept that fractionating the secretome enriches certain bioactive molecules involved in the repair and recovery of injured liver tissue.

**Graphical abstract:**

Exosome enriched mesenchymal stromal cell-derived fractionated secretome potentiates recovery upon injection in injured liver
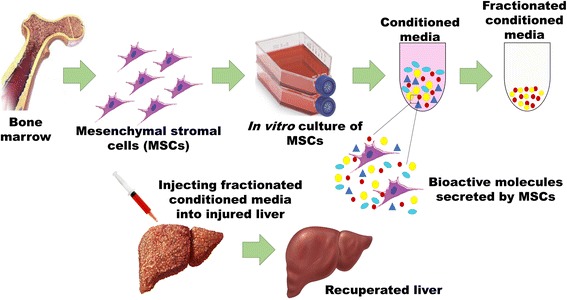

**Electronic supplementary material:**

The online version of this article (10.1186/s13287-017-0752-6) contains supplementary material, which is available to authorized users.

## Background

The liver has a remarkable capacity to self-heal or regenerate—a feature presumably evolved over the years to protect the liver from the catastrophic consequences of liver loss caused by food toxins [1]. Regeneration by the proliferation of the existing mature cellular population is a defense mechanism specific to the liver, triggered by injury conditions associated with drug/alcohol abuse, viral hepatitides and/or metabolic disorders [2–4]. Acute liver failure (ALF) occurs when the regenerative potential of the liver is overwhelmed until the rate of injury exceeds the rate of repair, and often results in death [5]. Liver transplantation is the only successful treatment mode for ALF. A shortage of organ donors [6] has led to the advent of therapeutic agents that can prevent further damage to the injured liver and stimulate the remnant liver cells to regenerate.

Mesenchymal stromal cells (MSCs) have been used in regenerative medicine due to their abundant expansion capacity and diverse differentiation potential [7]. Transplanted MSCs, however, do not necessarily engraft and differentiate at the site of injury. Even if engraftment does occur, it has been observed that the MSCs begin to transform into collagen-producing fibrocytes upon induction of chronic injury, preventing them from maintaining an epithelial-like characteristic [8]. Many studies have emphasized the role of immunomodulation and trophic effects in the therapeutic activity of MSCs [9]. It is elucidated that tissue injury stimulates the MSCs to secrete trophic factors collectively termed the MSC secretome. Paracrine signaling via extracellular vesicles present in the secretome may be responsible for mediating recovery from tissue injury [10, 11].

Studies in models of acute kidney failure [12, 13] have shown the potential of the MSC secretome to reduce/inhibit cell death and promote regeneration. Timmers and co-workers have shown the cardio-protective effects of an exosome-rich fraction of the MSC secretome explicating that the fraction may be enriched with soluble factors conferring the cardio-protective effects [14].

Cellular therapy using MSCs has been explored as an alternative treatment strategy for liver failure [15]. Many studies have shown the ability of MSCs to reduce liver fibrosis and improve liver function [16]. Although hepatic differentiation of MSCs has been demonstrated in vitro [17], it still remains a controversial point to prove in vivo [18, 19]. Hence, many reports propose the role of paracrine signaling by MSCs in the alleviation of liver failure conditions. Several studies have hinted at the role of chemokines and trophic factors released by MSCs in reducing tissue inflammation, cellular apoptosis and liver fibrosis, thus contributing to the overall improvement of liver function [20, 21].

In this work, we study the ability of extracellular vesicles present in the fractionated MSC secretome to attenuate liver injury conditions. First, conditioned medium collected from cultured rat bone marrow MSCs was fractionated using differential centrifugation and characterized for the presence of nanometer-sized extracellular vesicles known as exosomes. The effects of the medium were then studied in in-vitro models of liver injury in both two-dimensional (2D) and three-dimensional (3D) culture conditions. The results obtained were further validated in in-vivo models of acute liver injury.

## Methods

Except where noted, all materials used were procured from Sigma Aldrich, USA.

### Animals

Male Wistar rats (250–350 g) were used for isolation of bone marrow-derived MSCs and development of ALF models as per approval from the Institute Animal Ethics Committee (IITK/IAEC/2014/1023 and IITK/IAEC/2014/1022, respectively) of IIT Kanpur, under the Committee for the Purpose of Control and Supervision of Experiments on Animals (CPCSEA), Government of India. All methods were performed in accordance with relevant guidelines and regulations of this committee. The animals were housed in a climate-controlled environment with alternate 12-h light and dark cycles and having free access to standard food and water.

### Culture and seeding of HepG2 cells

HepG2 cells were cultured in Dulbecco’s modified Eagles’ medium (DMEM) supplemented with 10% fetal bovine serum (FBS) (Invitrogen, USA) and 1% antibiotic (HiMedia, India). The cells were passaged at 70% confluency with media changes every alternate day. For cell seeding, disc-shaped poly(*N*-isopropylacrylamide)-chitosan cryogels synthesized using a method described previously [22, 23] (height 2 mm; diameter 8 mm) were sterilized [22] and cells seeded at a density of 1.5 x 10^5^ cells/scaffold.

### Isolation and characterization of MSCs

Bone marrow-derived MSCs were isolated using a protocol described elsewhere with slight modifications [24]. The isolated cells were assessed for their ability to form colonies of spindle-shaped cells and potential to differentiate into osteogenic and adipogenic lineages. Detailed information for isolation and characterization of MSCs is provided in Additional file 1.

### Collection and characterization of exosome-rich fractionated secretome

**Fig. 1:**
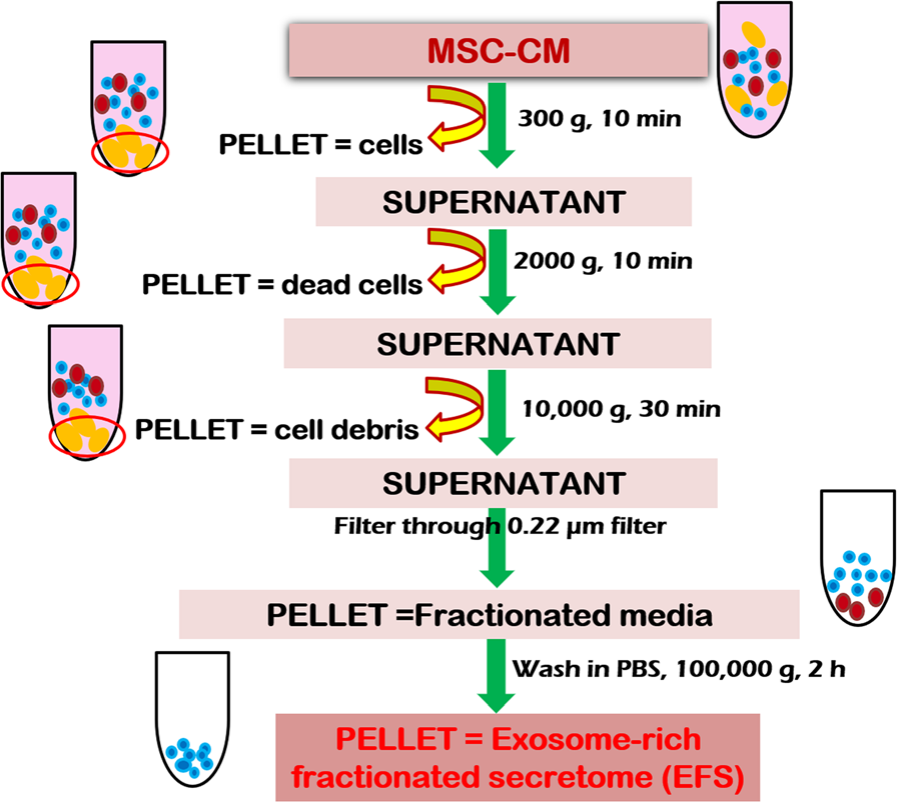
Schematic representation for generation of fractionated secretome. MSC mesenchymal stromal cell, CM conditioned medium, PBS phosphate buffered saline

Once the MSCs in passage 3 reached 70–80% confluency, complete medium was replaced with serum-free medium supplemented with 1% antibiotic and cells cultured for 48 h. After 48 h, the medium was collected and exosome-rich fractionated secretome (EFS) generated as depicted in Fig. 1. This EFS was further purified and concentrated using Amicon® Ultra Centrifugal filters (100 kDa MWCO) and the final protein concentration determined using BCA protein assay (Pierce, ThermoScientific, USA).

The fractionated secretome was characterized for the presence of exosomes. Dynamic light scattering (DLS) was performed using a Zetasizer series instrument (Malvern Instruments, United Kingdom) to analyze the size distribution of the exosomes. Confocal microscopy, scanning electron microscopy (SEM) and transmission electron microscopy (TEM) were used to visualize the vesicles using protocols established previously [25, 26].

Gel electrophoresis was used to confirm the enrichment of proteins in the exosomes vis-á-vis the protein content in the cell lysate. The fractionated secretome was evaluated for the presence of the exosomal biomarkers CD9, CD63 and CD81 using double sandwich ELISA assay (Exo-Test; HansaBiomed, Estonia), flow cytometry (Exo-FACS; HansaBiomed) and western blotting, respectively, according to the manufacturer’s protocols.

### Effect of exosome-rich fractionated MSC secretome on viability of liver cells

To check for cytotoxicity of the EFS, different concentrations of the secretome were incubated with HepG2 cells. The cells were seeded at a density of 1.5 × 10^4^ cells/well in complete medium supplemented with 0, 0.05, 0.1, 1, 10 and 50 μg/ml of fractionated secretome. Cell viability was monitored over 72 h using MTT assay [22].

### In-vitro study for recovery of viability in the presence of EFS

Two in-vitro models of liver injury were used to study the effect of EFS on the recovery of injured liver cells, namely treatment with 8 mM acetaminophen (APAP) and treatment with 60 μM hydrogen peroxide (H_2_O_2_).

HepG2 cells were seeded in tissue culture plates (2D) and in poly(*N*-isopropylacrylamide)-chitosan cryogel scaffolds (3D) at a density of 1.5 × 10^5^ cells/well or cells/scaffold. The cells were allowed to adhere for 24 h after which they were treated with 8 mM APAP/60 μM H_2_O_2_ in complete media supplemented with 0.5 μg/ml fractionated secretome or 0.5 μg/ml unfractionated secretome. The viability of the cells was monitored over 72 h quantitatively using MTT assay and lactate dehydrogenase (LDH) activity (as per the standard manufacturer’s protocol), and qualitatively using fluorescence microscopy (Additional file 1).

### Effect of EFS on ROS accumulation due to liver injury

A common phenomenon associated with liver injury is the release of reactive oxygen species (ROS), which was measured in vitro using 2′,7′-dichlorofluorescein diacetate (DCFDA) assay (Additional file 1).

### Rodent models of liver failure

#### Ischemic/reperfusion liver injury during partial hepatectomy

Partial hepatectomy (2/3^rd^) was performed using the method described by Higgins and Anderson with some modifications [42]. After making a mid-abdominal incision, the upper abdomen and lateral lower portions of both hemithoraces were compressed to partly exteriorize the liver. Further, the medial and left lateral lobes were tied down close to the hilum using a 2-0 cotton thread. Consequently, the hepatic portal vein (HPV) and hepatic artery (HA) were clamped for 30 min to create an ischemic condition. After 30 min, the knotted lobes were resected en bloc, the clamp on the HPV and HA removed, and the abdomen sutured closed using prolene sutures.

#### Acute liver injury using carbon tetrachloride

To establish acute liver injury, rats were given an intraperitoneal injection of carbon tetrachloride (CCl_4_) 20% (v/v) solution in olive oil at a dose of 5 ml/kg, corresponding to a single dose of 1 ml/kg. The animals were monitored for 1 week and blood samples were taken every 24 h.

### Effect of EFS on liver regeneration rate and recovery of liver functions in rodent models of liver failure

In the ischemic/reperfusion injury model, before removing the clamp, 50 μg of EFS in 100 μl phosphate buffered saline (PBS) was injected via the HPV in one group of animals (*n* = 5) and 50 μg of unfractionated secretome (reconstituted in PBS after lyophilizing) was injected in another group of animals (*n* = 5). The control group (*n* = 3) was not injected with any solution. The animals were monitored for 72 h since this is the period in which any effect on the response to injury can be studied closely. The total liver regeneration rate was calculated at the end of 72 h.

In the CCl_4_ model, 50 μg of EFS in 100 μl saline was injected via HPV 24 h post CCl_4_ injection (*n* = 5). The control group was injected with 100 μl PBS in the HPV (*n* = 3). The animals were monitored for 1 week. Three animals were sacrificed at 72 h post secretome injection and the livers harvested for histological evaluation using hemotoxylin and eosin (H&E). Immunofluorescence was carried out to check for expression of PCNA (PC10, sc-56, 1:100; Santa Cruz Biotechnology, Inc.) and 8-OHdG (8-OHdG (15A3), sc-66036, 1:100; Santa Cruz Biotechnology, Inc.) to assay hepatocyte proliferation and oxidative stress.

Blood samples were collected from both liver failure models every 24 h and analyzed for liver function parameters aspartate transaminase (AST), alanine transaminase (ALT), bilirubin and albumin using a blood biochemical analyzer (ERBA Mannheim, Germany).

### Statistical analysis

Statistical comparisons were evaluated using GraphPad Prism software. The differences between two groups were analyzed using Student’s *t* test.

## Results and discussion

### Characterization of MSCs

Typically, MSCs are characterized as plastic-adherent cells when maintained in standard culture conditions, forming clusters that eventually interconnect into a monolayer and the ability to differentiate into osteoblasts, adipocytes and chondroblasts in vitro. On isolation, the MSCs attached to the culture flasks and showed a spindle-shaped morphology as seen in the microscopic images of the MSCs (Additional file 2: Figure S1A, B). On culturing MSCs in a 100-mm Petri dish, the cells proliferated gradually into small colonies. Eventually, as the cells grew, adjacent colonies began to interconnect with each other and a confluent monolayer was observed using crystal violet staining at the end of 14 days (Additional file 2: Figure S1C, D).

When assayed for osteogenic differentiation, whereas the cells cultured in complete medium showed no visible difference in morphology (Additional file 2: Figure S1E), cellular aggregates were observed in the cells treated with osteogenic differentiation medium. Further, the cells stained with Alizarin Red (Additional file 2: Figure S1F), indicating extensive calcium deposition—a typical indication of osteogenic differentiation. Similarly, when assayed for adipogenic differentiation, the cells transformed in morphology, taking up a more ovoid shape with lipid droplets accumulating in the cellular periphery. Oil Red O stained these lipid droplets in the differentiated cells. No such staining occurred in the control cells (Additional file 2: Figure S1G, H).

The isolated cells may be characterized as MSCs, since they demonstrate the ability to adhere to plastic tissue culture flasks, form clusters of cells with a fibroblast-like morphology and can differentiate into osteogenic and adipogenic lineages.

### Characterization of exosome-rich fractionated secretome

**Fig. 2:**
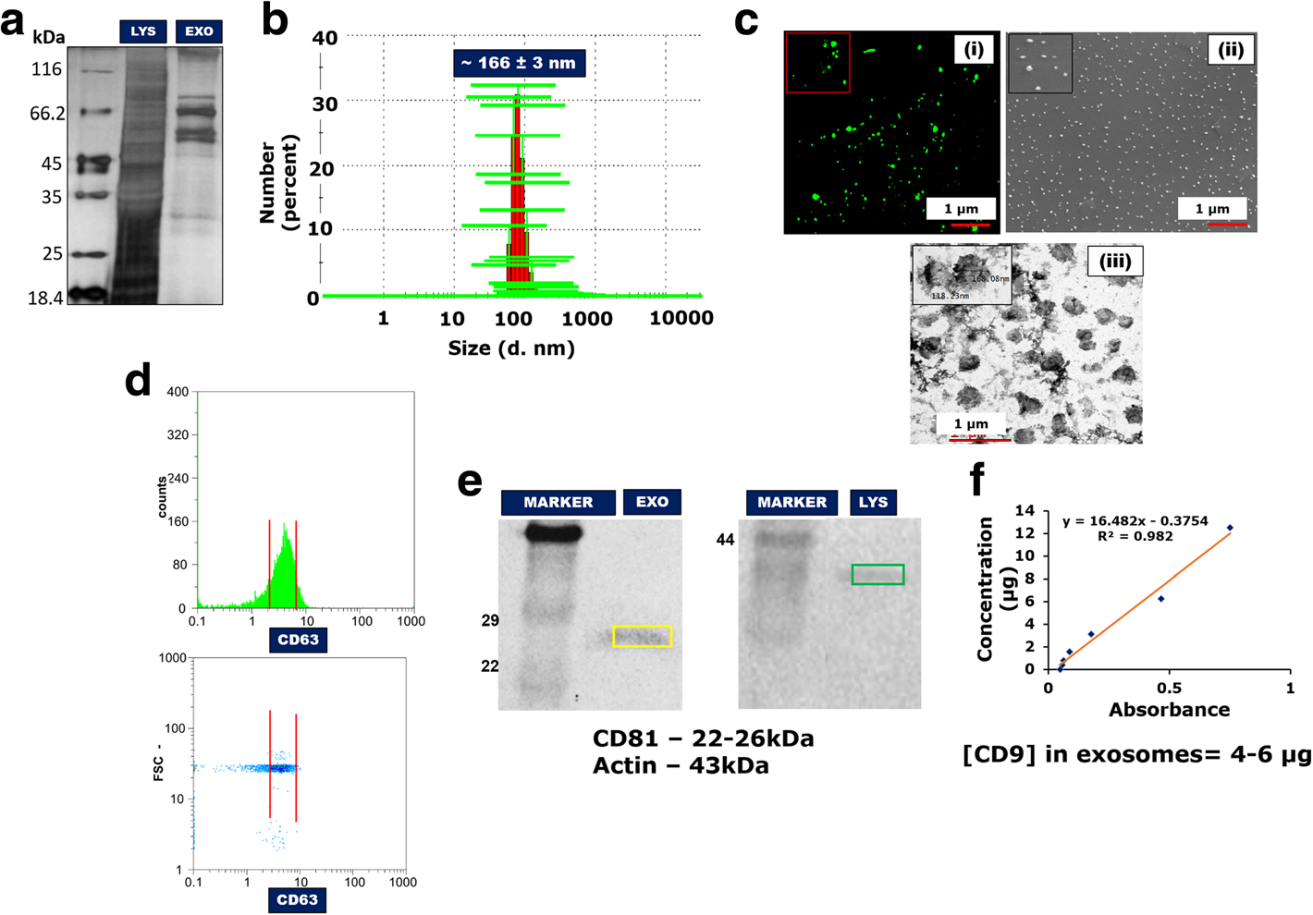
Characterization of MSC fractionated secretome. SDS-PAGE gel for MSC cell lysate (LYS) and exosome-rich fractionated MSC secretome (EXO) (**a**). DLS data showing average *z*-value representative of average particle size (**b**). Confocal microscopic image at lower (×20) and higher (×100) magnification (inset) (scale bar: 1 μm) (**c**): (i) scanning electron microscopic image (scale bar: 1 μm), (ii) transmission electron microscopic image (scale bar: 1 μm) and (iii) for EFS. Flow cytometric analysis for presence of exosomal marker CD63 in the fractionated secretome (**d**). Western blot analysis showing presence of exosomal marker CD81 (**e**). Standard curve for sandwich ELISA performed to confirm presence of exosomal marker CD9 in the fractionated MSC secretome (**f**). FSC forward scatter

MSC secretome has been known to contain several molecules including, but not limited to, growth factors, hormones, metabolites, ions, polysaccharides and proteins [27–29]. Targeted approaches have identified many proteins present in the MSC secretome that could possibly play a role in its therapeutic activity [27]. The protein content of the concentrated EFS was found to be approximately 270 μg/ml. Gel electrophoresis using SDS-PAGE showed the EFS to contain fewer protein bands compared to the cell lysate, indicating that it may contain a subset of proteins present in the cell lysate (Fig. 2a).

#### DLS, confocal microscopy, SEM and TEM

**Fig. 3:**
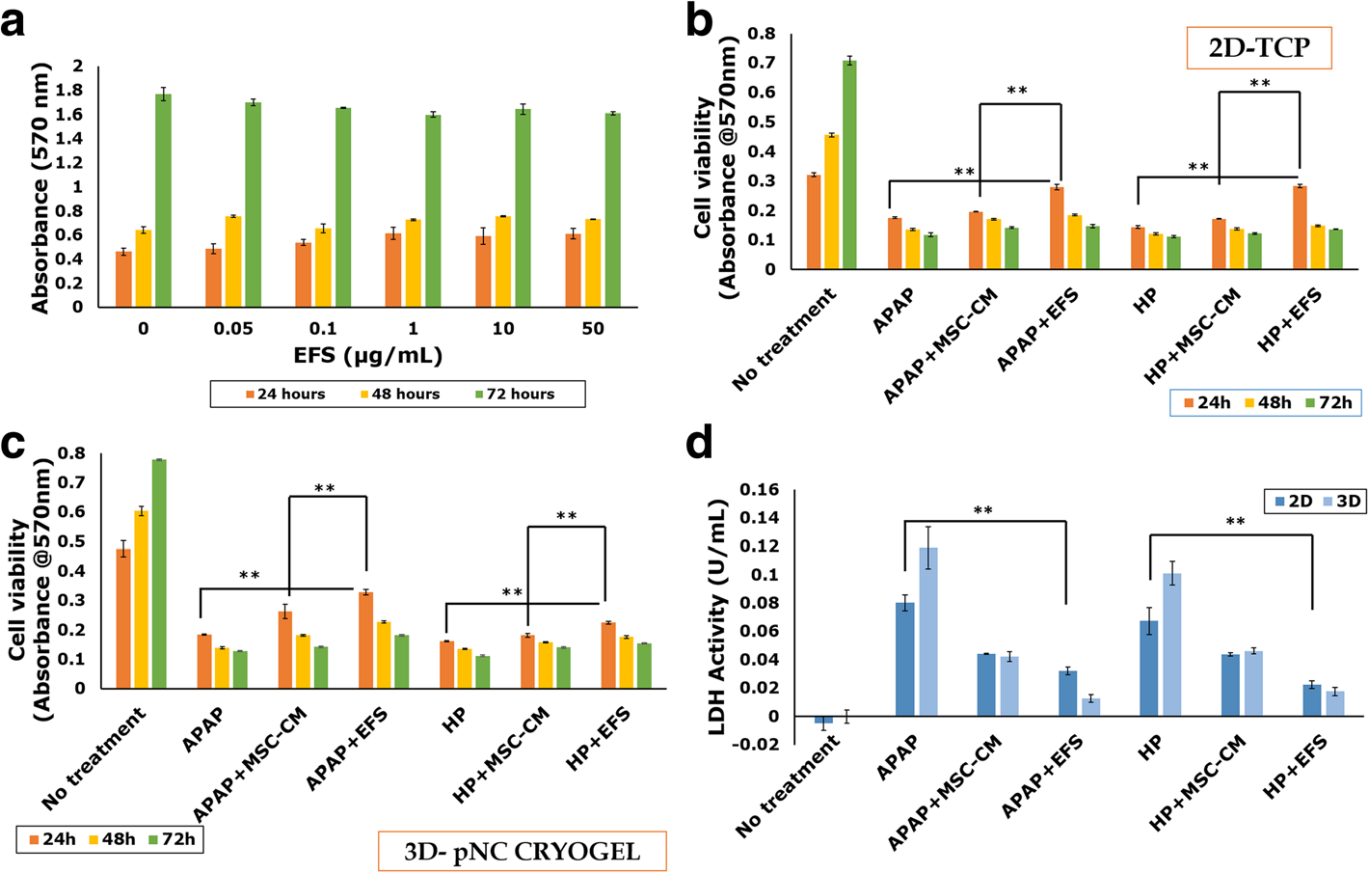
Effect of EFS on viability of liver cells. Effect of different concentrations of exosome-rich fractionated secretome (EFS) on viability of HepG2 cells over a period of 72 h (**a**). MTT assay to check effect of fractionated secretome and unfractionated secretome on viability of injury-induced liver cells in 2D tissue culture plate (2D-TCP) (**b**) and 3D cryogel scaffold (3D-pNC CRYOGEL) (**c**). LDH activity of acetaminophen (APAP) and hydrogen peroxide (HP) injury-induced liver cells before and after treatment with unfractionated MSC secretome (MSC-CM) and fractionated MSC secretome (EFS) (**d**). Statistical analysis: *n* = 3, **p < 0.01. 2D two dimensional, 3D three dimensional, MSC mesenchymal stromal cell, CM conditioned medium, LDH lactate dehydrogenase

In addition to the large milieu of soluble factors present in the secretome, there have been several reports on the presence of extracellular vesicles (EVs) in the MSC secretome [30, 31]. One type of EVs found in the MSC secretome are the exosomes, nanometer-sized membrane vesicles (~30–120 nm) formed by the inward budding of multivesicular bodies (MVB). They are involved in the trafficking and transfection of bioactive molecules to and from cells, enabling cell–cell communication [32]. It has been increasingly recognized that these exosomes could be responsible for conferring the secretome its therapeutic potential [31]. Dynamic light scattering (DLS) was used to identify the presence of exosomes in the fractionated secretome. The *z*-value/harmonic intensity averaged particle diameter of the fractionated secretome was 165.98 ± 3.16 nm (polydispersity index = 0.324 ± 0.026) (Fig. 3b), suggesting the presence of nanometer-sized vesicles. This was further confirmed by confocal microscopy (Fig. 2c i), SEM (Fig. 2c ii) and TEM (Fig. 2c iii), which showed the presence of vesicular structures in the fractionated secretome.

#### Flow cytometry, western blotting and sandwich ELISA

The presence of endosomal membrane markers CD9, CD63 and CD81 typically characterizes exosomes [25, 32, 33]. The presence of the CD63 marker was confirmed by flow cytometric analysis (Fig. 2d), whereas western blot assay confirmed the presence of CD81 (Fig. 2e). Furthermore, sandwich ELISA confirmed the presence of CD9 (Fig. 2f). The presence of all three markers in the fractionated secretome ascertains the presence of exosomes in the fraction.

### Fractionated secretome does not have any cytotoxic effect on liver cells

Different protein concentrations of the EFS were tested on HepG2 cells, to assess its cytotoxicity. HepG2 cells were seen to maintain their viability at different concentrations, suggesting that the fraction was not cytotoxic (Fig. 3a). For further in-vitro studies a concentration of 0.5 μg/ml was used, whereas a concentration of 50 μg/ml was used for the in-vivo studies.

### Exosomes in the fractionated secretome confer a cytoprotective effect on liver cells in in-vitro models of liver injury

To study the effect of exosomes in the fractionated secretome on liver cells in the case of liver injury, in-vitro models were designed using APAP and H_2_O_2_. The APAP-induced liver injury model is representative of the covalent modification of protein targets as well as the oxidative stress pathway typical of the pathogenesis of liver failure in vivo [34, 35]. Likewise, H_2_O_2-_induced liver injury closely mimics the oxidative stress generated during early phases of liver injury in vivo [36].

**Fig. 4:**
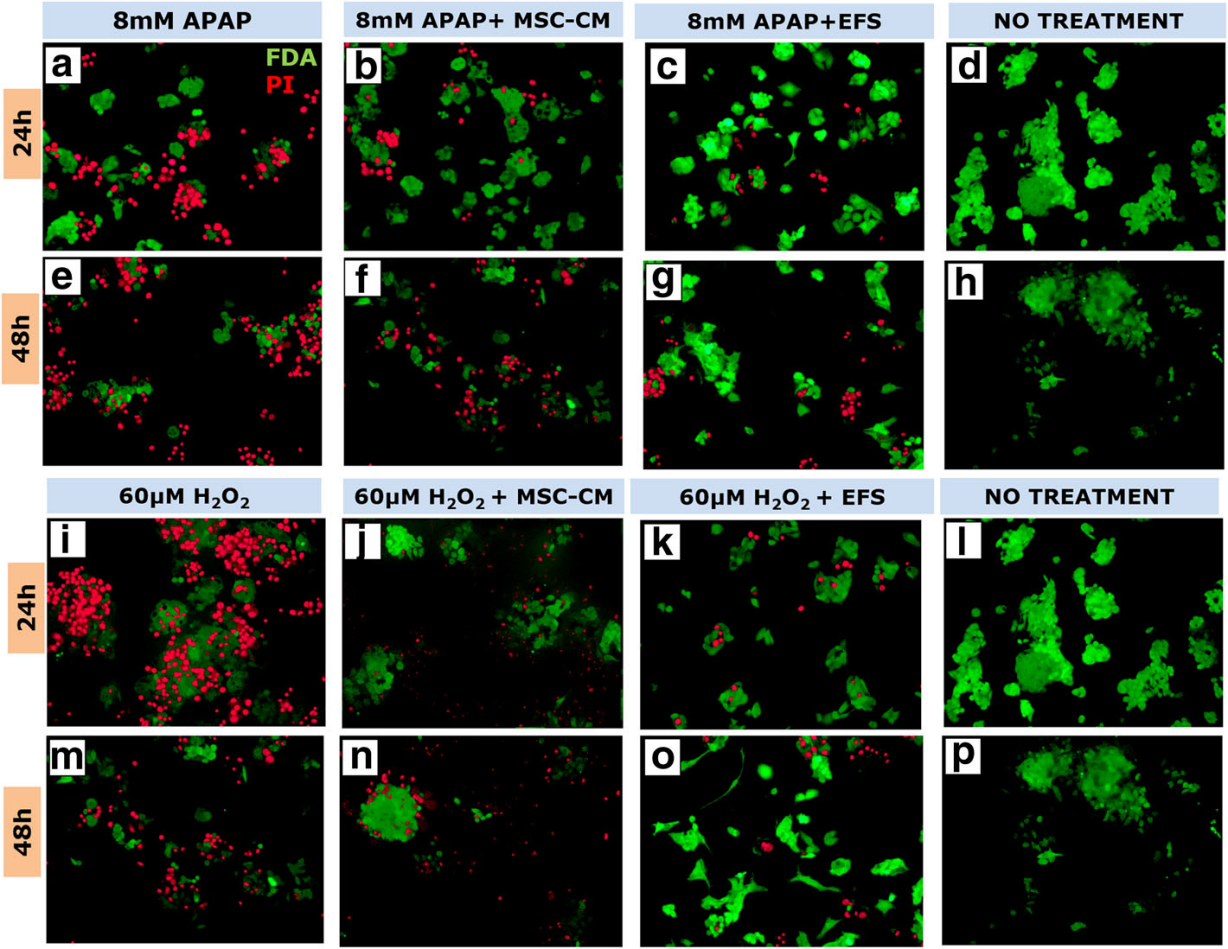
Fluorescent microscopic analysis of the effect of EFS on liver cell viability in injury conditions. Live–dead staining using FDA and PI 24 and 48 h post treatment with 8 mM acetaminophen (APAP) (**a** and **e**, respectively), 8 mM APAP in presence of unfractionated MSC secretome (**b** and **f**, respectively), 8 mM APAP in presence of fractionated MSC secretome (**c** and **g**, respectively) and without any treatment (**d** and **h**, respectively). Live–dead staining using FDA and PI 24 and 48 h post treatment with 60 μM hydrogen peroxide (H_2_O_2_) (**i** and **m**, respectively), 60 μM H_2_O_2_ in presence of unfractionated MSC secretome (**j** and **n**, respectively), 60 μM H_2_O_2_ in presence of fractionated MSC secretome (**k** and **o**, respectively) and without any treatment (**l** and **p**, respectively). Scale bar for all microscopic images: 100 μm. MSC mesenchymal stromal cell, CM conditioned medium, EFS exosome-rich fractionated secretome

It was observed that the EFS conferred better cytoprotective effect as compared to the unfractionated secretome in APAP and H_2_O_2_-induced liver injury models in 2D tissue culture plates (Fig. 3b) as well as in 3D cryogel scaffolds (Fig. 3c). Further, the LDH activity 24 h after injury induction shows a significant decrease in the cells treated with fractionated as compared to unfractionated secretome or untreated cells (Fig. 3d). These results collectively suggest the presence of some factors in the enriched fraction which are involved in protecting the liver cells from necrosis and/or apoptosis. This cytoprotective effect of the EFS was also observed in the microscopic studies wherein fewer cells were stained with PI in the presence of fractionated secretome as compared to unfractionated secretome (Fig. 4).

### Exosomes in the fractionated secretome reduce the oxidative stress generated due to injury conditions

Oxidative stress is a major pathogenic phenomenon characteristic of liver disorders and a major cause of liver damage due to ischemic/reperfusion during liver transplantation [37]. Normally, liver cells are equipped to control the level of oxidative stress and maintain a balance between oxidant and antioxidant particles. However, upon damage due to toxin-induced injury, there is an imbalance between these particles. Oxidative stress occurs due to mitochondrial dysfunction of the liver cells that leads to a continuous increase in ROS production, which not only induces irretrievable alterations of lipids, proteins and DNA contents but also modulates pathways that control normal biological functions [38].

**Fig. 5:**
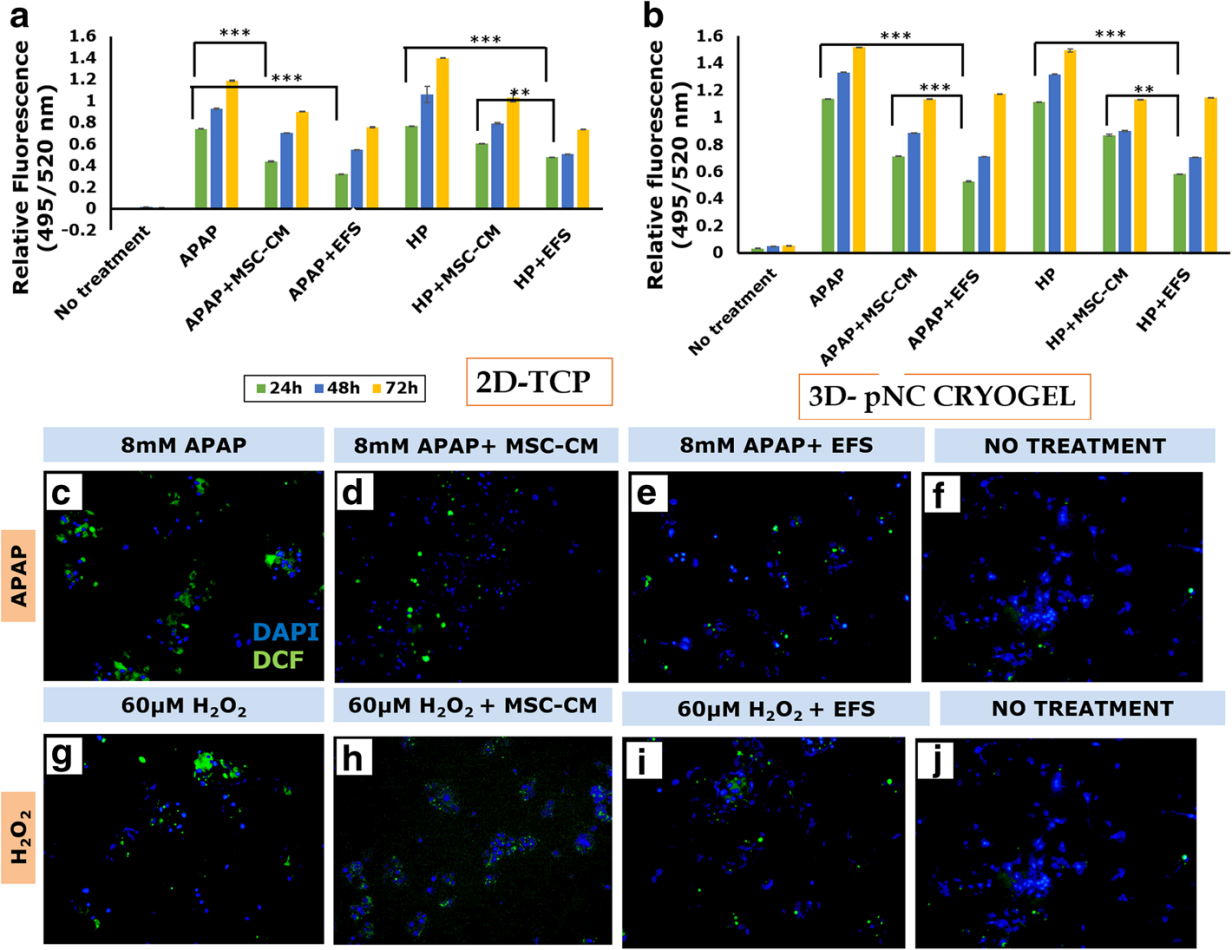
Effect of EFS on oxidative stress induced during liver injury. Quantitative DCFDA assay for reactive oxygen species (ROS) released by liver cells post treatment with acetaminophen (APAP) and hydrogen peroxide (HP) in the presence of unfractionated MSC secretome (MSC-CM) and fractionated MSC secretome (EFS) in 2D tissue culture plate (2D-TCP) (**a**) and 3D cryogel scaffold (3D-pNC CRYOGEL) (**b**). Qualitative DCFDA assay for ROS 24 h post treatment with 8 mM APAP (**c**), 8 mM APAP in presence of unfractionated MSC secretome (**d**), 8 mM APAP in presence of fractionated MSC secretome (**e**) and without any treatment (**f**). Qualitative DCFDA assay for ROS 24 h post treatment with 60 μM hydrogen peroxide (H_2_O_2_) (**g**), 60 μM H_2_O_2_ in presence of unfractionated MSC secretome (**h**), 60 μM H_2_O_2_ in presence of fractionated MSC secretome (**i**) and without any treatment (**j**). Scale bar for all microscopic images: 100 μm. Statistical analysis: *n* = 3, **p < 0.01, ***p < 0.001. MSC mesenchymal stromal cell, CM conditioned medium, EFS exosome-rich fractionated secretome, 2D two dimensional, 3D three dimensional

Both APAP and H_2_O_2_-induced liver injury result in the formation of large amounts of ROS which eventually lead to cell death. It was observed that the EFS could reduce ROS activity in injury-induced liver cells significantly better than the unfractionated secretome (Fig. 5). MSCs are known to have high levels of glutathione (an antioxidant) and an enhanced ROS-scavenging property [39]. Additionally, recent studies have shown the potential of vesicles present in the MSC secretome to reduce oxidative stress via peroxiredoxins and glutathione S-transferases present in them [40]. Hence, the fraction generated via ultracentrifugation of the MSC conditioned media could have enriched these vesicles containing antioxidant particles, enabling the fractionated secretome to reduce oxidative stress on the injury-induced liver cells.

It was further observed that ROS activity in the cells cultured on 3D cryogel scaffolds was relatively higher as compared to that of cells cultured in 2D, despite the antioxidative effect of the fractionated secretome (Fig. 5a, b). Reports have shown that hepatic transporters play a crucial role in the import of xenobiotic substances and efflux of their metabolites by various hepatic enzymes [41]. The localization and functionality of these transporters is largely affected by cell culture conditions and an increase in transporter activity is observed in 3D culture [41], hence the increase in ROS activity in 3D culture. Interestingly, however, the cell viability studies discussed show a higher cell viability in 3D culture treated with fractionated secretome, despite the high ROS activity. This suggests that the increase in cell viability may not necessarily be just because of the antioxidative effect of the fraction but due to a synergistic effect involving both antioxidative and prosurvival effects of the trophic factors enriched in the EFS.

### Exosome-rich fractionated secretome improves liver regeneration and recovery of liver functions in vivo

To validate our results from in-vitro studies, we used two models for liver failure. Partial hepatectomy is the classical model for liver failure/regeneration. This model was further modified to include ischemic/reperfusion injury, a common clinical phenomenon during hepatic resection and liver transplantation that frequently leads to surgical failure of the transplant due to hypoxic cellular damage [38]. The classical technique of 70% partial hepatectomy [42] is associated with a lot of intraoperative blood loss. Hence the Pringle maneuver technique [43] of clamping the HPV and HA was used to reduce the risk of bleeding from the stump and induce ischemic injury. In our model, the HPV and HA were clamped for 30 min to induce ischemic injury.

**Fig. 6:**
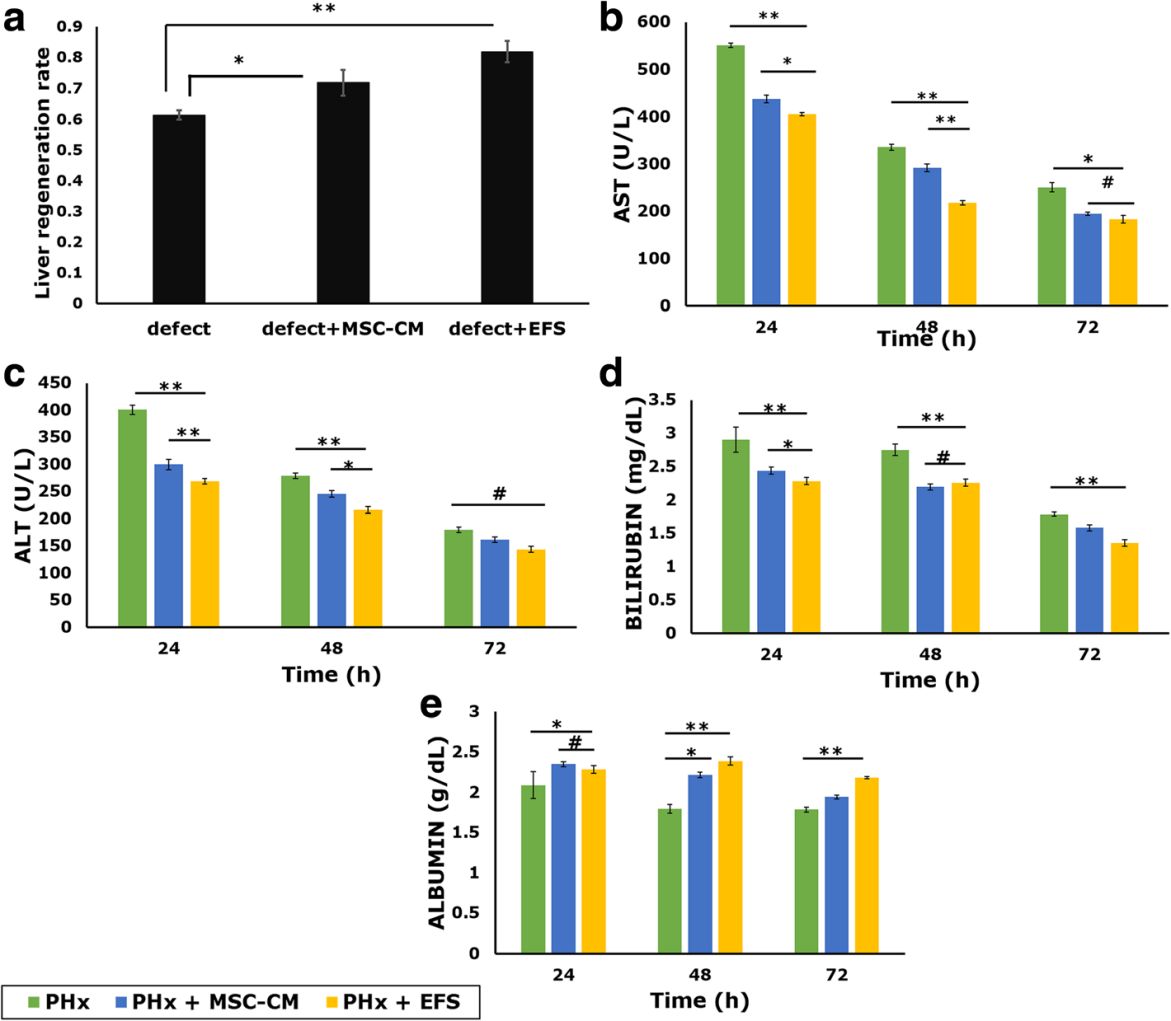
Effect of EFS on liver regeneration and recovery in ischemic/reperfusion model of liver failure. Liver regeneration rate of untreated rodent model (defect), model treated with unfractionated MSC secretome (defect + MSC-CM) and model treated with fractionated MSC secretome (defect + EFS). Statistical analysis: *n* = 3 (control); *n* = 5 (models), *p < 0.05, **p < 0.01 (**a**). AST (**b**), ALT (**c**), bilirubin (**d**) and albumin (**e**) levels in untreated liver failure models (PHx), models treated with unfractionated secretome (PHx + MSC-CM) and models treated with fractionated secretome (PHx + EFS). Statistical analysis: *n* = 3, *p < 0.05, **p < 0.01, #ns. MSC mesenchymal stromal cell, CM conditioned medium, EFS exosome-rich fractionated secretome, AST aspartate transaminase, ALT alanine transaminase, PHx partial hepatectomy

The liver regeneration rate, calculated as the ratio of the remnant liver weight to the estimated liver weight, was found to be highest in the group injected with EFS (0.819 ± 0.035), followed by the group injected with unfractionated secretome (0.718 ± 0.042). Both of these values were higher than the regeneration rate of the untreated group (0.614 ± 0.15) (*p* < 0.01 and *p* < 0.05, respectively) (Fig. 6a).

Earlier reports have shown that the values of AST and ALT increase within the first few hours of liver injury, indicating cytotoxicity to the liver cells. As the liver recuperates and regeneration sets in, AST/ALT levels reduce with time. In our study, although elevated, lower levels of AST and ALT were observed after 24 h in the groups injected with EFS (26.4% and 33%, respectively) and unfractionated secretome (20.5% and 25%, respectively) as compared to the untreated group. Further, both AST and ALT were lower in the group treated with EFS as compared with unfractionated secretome (Fig. 6b, c). Also, the level of bilirubin in the untreated animal model was found to be significantly higher than that in the unfractionated secretome, which was still higher than that in the EFS (Fig. 6d). Albumin levels, although reduced, were significantly higher in the treated groups as compared with the untreated models (Fig. 6e). These results suggest once again that the fraction contains factors responsible for alleviating liver injury conditions.

Carbon tetrachloride intoxication is a commonly used model for both acute and chronic liver injury [44]. The short-term administration of CCl_4_ causes hepatic injury, primarily through apoptosis and necrosis of the hepatic cells [45], with a majority of the cell death taking place in the centrilobular region. Since the human liver metabolizes CCl_4_ in a similar manner to that in rodents, CCl_4_-induced liver injury is an appropriate model for chemical-induced liver injury [46].

**Fig. 7:**
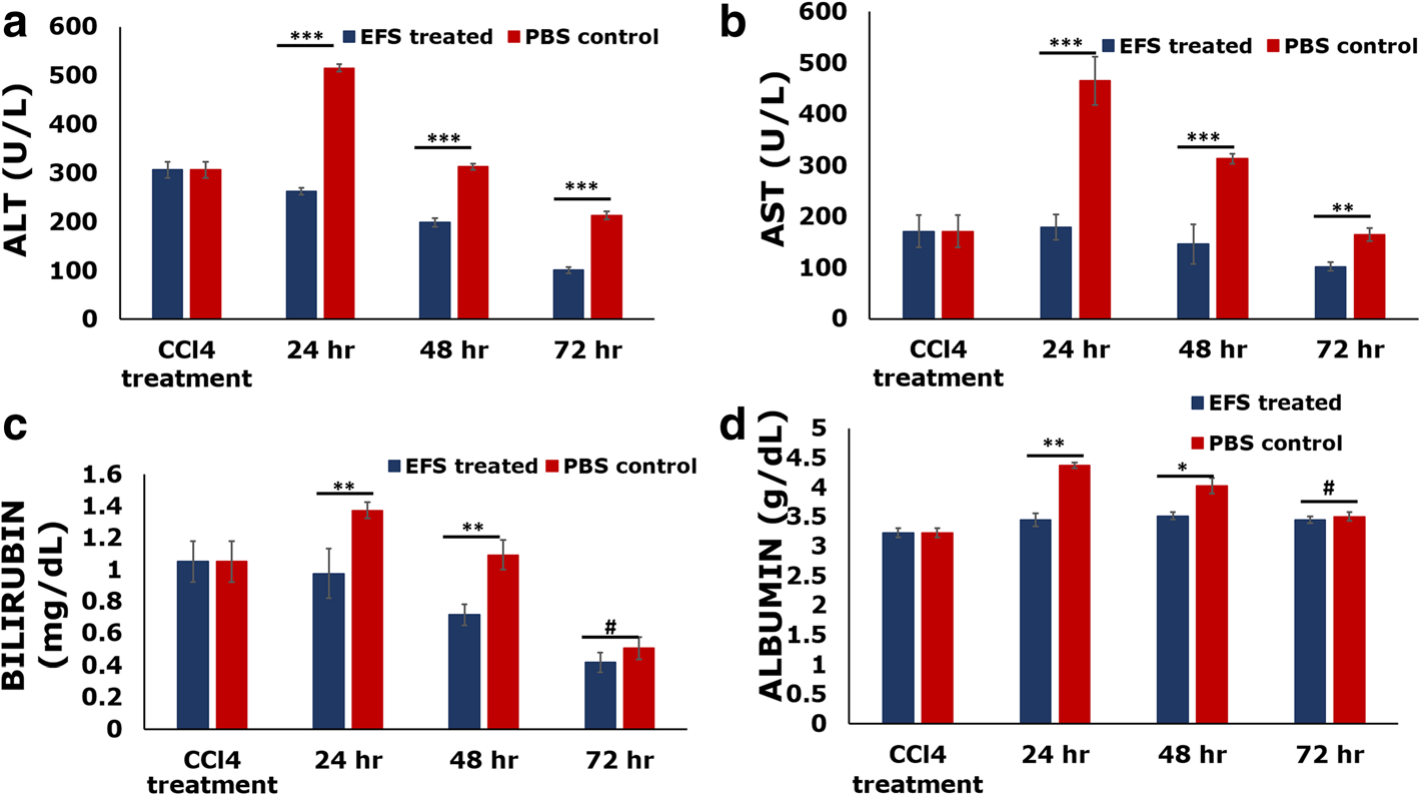
Effect of EFS on recovery in carbon tetrachloride-induced liver injury model. ALT, AST, bilirubin and albumin levels in liver failure model before treatment (CCl_4_ treated) and CCl_4_ models treated with EFS (EFS treated) and treated with PBS (PBS control) at regular time intervals post injection. Statistical analysis: *n* = 3, *p < 0.05, **p < 0.01, ***p < 0.001, #ns. AST aspartate transaminase, ALT alanine transaminase, CCl4 carbon tetrachloride, EFS exosome-rich fractionated secretome, PBS phosphate buffered saline

CCl_4_ challenge results in elevated serum levels of ALT and AST [47]. In our study, upon administration of CCl_4_ the levels of ALT and AST shoot up to almost 2-fold the normal values. Injection of EFS in the HPV 24 h post CCl_4_ injection results in a significant decrease in the levels of ALT (~67%) and AST (~40%) over a period of 72 h as compared to models injected with PBS (~30% and ~ 5%, respectively). The levels of both ALT and AST remain significantly lower in the EFS injected models as compared to the PBS injected models (Fig. 7a, b). A similar trend was observed for the levels of bilirubin (Fig. 7c). Although there was no significant change in the levels of albumin before and after CCl_4_ administration, the levels were significantly lower in the EFS injected models as compared to the PBS injected models (Fig. 7d).

**Fig. 8:**
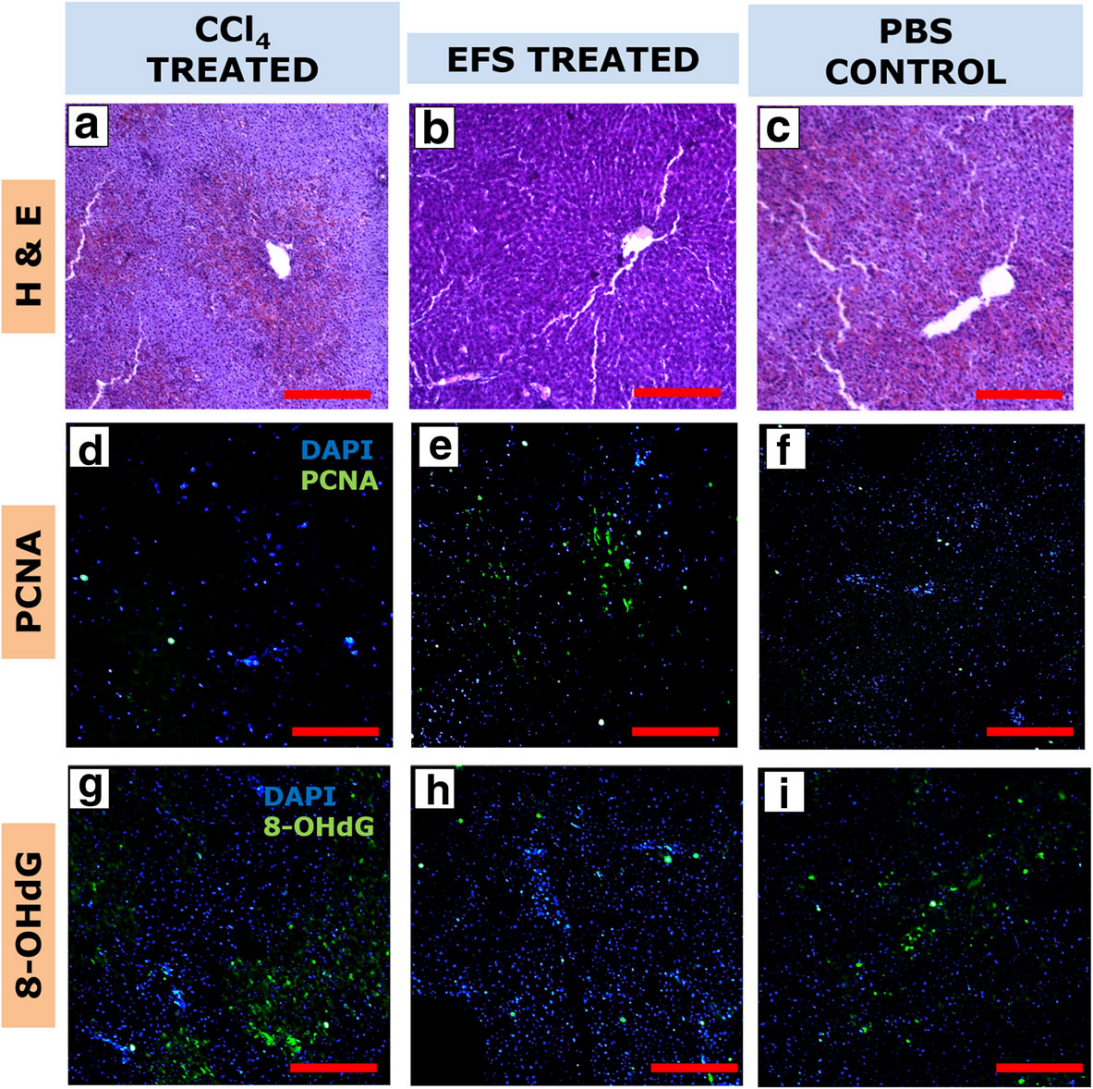
Effect of EFS on hepatocyte proliferation and oxidative stress in carbon tetrachloride-induced liver injury. Hematoxylin and eosin (H&E) staining (**a–c**), PCNA expression (**d–f**) and 8-OHdG expression (**g–i**) of untreated (CCl_4_ treated), EFS (EFS treated) and PBS treated (PBS control) injury models 72 h post injection. Scale bar for all microscopic images: 100 μm. *CCl*_4_ Carbon tetrachloride; *EFS* Exosome-rich fractionated secretome; *PBS* Phosphate buffered saline; *PCNA* Proliferating cell nuclear antigen; *8-OHdG* 8-hydroxy-2' -deoxyguanosine; *DAPI* 4',6-diamidino-2-phenylindole

The effects on the biochemical parameters were further corroborated by histological analysis of liver tissue collected 72 h post treatment with exosomes. H&E staining revealed cell death across the liver tissue in the untreated CCL_4_-induced models (Fig. 8a). Sections of tissues collected from models treated with exosomes post CCl_4_ intoxication showed a significantly reduced amount of cell death (Fig. 8b) as compared to control animals which were treated with PBS (Fig. 8c). The administration of EFS to CCl_4_-treated rats was found to confer a cytoprotective effect with increased proliferation of hepatocytes, indicated by increased expression levels of PCNA as compared to untreated models and models treated with PBS (Fig. 8d–f). In addition, decreased expression of 8-OHdg, a product of oxidative stress in the liver cells, in the models treated with EFS vis-á-vis untreated and PBS treated, confirms the antioxidative effect of the fraction which was established through in-vitro studies (Fig. 8 g–i).

Recent studies on the proteomic analysis of the MSC exosomes reveal the enrichment of more than 200 proteins [48–50]. These enriched proteins have been shown to be functionally linked to important biological processes such as angiogenesis, blood coagulation, apoptosis, regulation of inflammation and extracellular matrix remodeling [48, 51]. Furthermore, a detailed study on the mRNA and miRNA cargo of the MSC exosomes reveals the potential of these RNAs to regulate the transcription of genetic information as well as modulate angiogenesis, extracellular matrix turnover and TGF-β signaling [49].

MSCs have been shown to be involved in immune modulation [52]. A detailed analysis of the MSC-derived exosomes shows their potential to be immunologically active with an increased expression of inflammatory mediators such as interleukin 10 (IL-10) and attenuated levels of IL-6 and TNF-α [53]. Some studies have shown the prominent role of TNF-α and IL-6 in initiating liver regeneration, with their levels increasing on injury [54, 55]. IL-6 has a hepatoprotective effect on hepatocytes and its increased secretion can be induced by the IL-6 present in the MSC exosomes [56]. On the other hand, TNF-α in the liver along with TNF-α in the MSC exosomes may help improve regeneration by reducing the inflammatory response typical to any form of tissue injury. Furthermore, other related studies have also shown the copurification of a functional ‘immunoproteasome’ within the MSC exosomes that seems to confer the tissue repair effect associated with the MSC exosomes [50].

As mentioned earlier, proteomic analysis of the MSC exosomes reveals their involvement in extracellular matrix remodeling. A number of the proteins identified in the exosomes are associated with ECM turnover. Of these, the matrix metalloproteinase 9 (MMP9) seems to be found in abundance in the exosomes [49]. The MMP9 enzyme is involved in extracellular matrix breakdown and could be involved in the attenuation of liver injury by reducing the amount/progression of fibrosis resulting from liver injury [49, 57]. Studies on the effect of MSC exosomes on scar formation and wound healing have shown the ability of the exosomes to prevent the differentiation of fibroblasts to myofibroblasts as well as to promote ECM reconstruction by regulating the levels of collagen III to collagen I, TGF-β3 to TGF-β1 and MMP3 to TIMP1 [58]. In the context of liver diseases, exosomes from human umbilical cord blood-derived MSCs showed a significant decrease in the concentrations of collagen I/III and TGF-β1 as well as reduced phosphorylation Smad2 expression in a carbon tetrachloride-based liver fibrosis model [59]. A similar effect on the collagen I/III and TGF-β1 levels may be responsible for the ‘reparative’ effect of the exosomes observed in our study.

## Conclusion

Bone marrow-derived MSC-conditioned medium was fractionated using ultracentrifugation. The fractionated secretome was found to be enriched with exosomes that confer antiapoptotic and/or prosurvival effects as well as antioxidative effects in in-vitro models of liver injury and improved liver regeneration and recovery from liver injury in vivo. Further proteomic analysis as well as gene expression studies need to be carried out to elucidate the mechanism by which the EFS exerts its therapeutic potential. However, the study provides a proof of concept that fractionating the secretome enriches exosomes/trophic factors involved in improving liver regeneration. Exosome-rich fractionated secretome may be used clinically in liver transplantation cases to reduce ischemic/reperfusion injury-related tissue damage or in the treatment of acute liver failure.

## Additional files

Additional file 1: presents additional information on methods to isolate and characterize MSCs; how to characterize the exosome-rich fractionated secretome using microscopy, flow cytometry, ELISA and western blot techniques; quantifying ROS activity in cells; performing qualitative fluorescence microscopy analysis for the in-vitro experiments; and calculation of the liver regeneration rate

Additional file 2: Figure S1. showing characterization of rat bone marrow-derived mesenchymal stem cells. Phase-contrast microscopic image of cultured MSCs showing fibroblast-like spindle-shaped morphology (**A**), fluorescence microscopic image of MSCs stained with fluorescein diacetate (FDA) (**B**), digital image of crystal violet staining of colonies of MSCs formed (**C**), microscopic image of MSCs stained with crystal violet (**D**), Alizarin Red staining of undifferentiated MSCs (**E**) and osteogenically differentiated MSCs (**F**) and Oil Red O staining of undifferentiated MSCs (**G**) and adipogenically differentiated MSCs (**H**). Scale bar for all microscopic images: 100 μm (TIF 2112 kb)

## Abbreviations

2D: Two dimensional
3D: Three dimensional
ALF: Acute liver failure
ALT: Alanine transaminase
APAP: Acetaminophen
AST: Aspartate transaminase
DCFDA: 2′7′-dichlorofluroscein diacetate
DLS: Dynamic light scattering
DMEM: Dulbecco’s modified Eagles’ medium
ECM: Extracellular matrix
EFS: Exosome-rich fractionated secretome
EV: Extracellular vesicle
FBS: Fetal bovine serum
H&E: Hematoxylin and eosin
HA: Hepatic artery
HPV: Hepatic portal vein
IL: Interleukin
LDH: Lactate dehydrogenase
MMP: Matrix metalloproteinase
MSC: Mesenchymal stromal cell
MTT: 3-(4,5-Dimethylthiazol-2-yl)-2,5-diphenyltetrazolium bromide
MVB: Multivesicular bodies
MWCO: Molecular weight cutoff
PBS: Phosphate buffered saline
PHx: Partial hepatectomy
ROS: Reactive oxygen species
SEM: Scanning electron microscopy
TEM: Transmission electron microscopy
TGF: Transforming growth factor
TNF: Tumor necrosis factor
