# Supplementary material for: Mesenchymal stromal cell-derived exosome-rich fractionated secretome confers a hepatoprotective effect in liver injury
Source: Stem Cell Res Ther. 2018 Feb 6;9:31. doi: 10.1186/s13287-017-0752-6 (PMC5801895; doi:10.1186/s13287-017-0752-6)
Supplement: Supplementary file 1 — presents additional information on methods to isolate and characterize MSCs; how to characterize the exosome-rich fractionated secretome using microscopy, flow cytometry, ELISA and western blot techniques; quantifying ROS activity in cells; performing qualitative fluorescence microscopy analysis for the in-vitro experiments; and calculation of the liver regeneration rate [file 13287_2017_752_MOESM1_ESM.pdf]

Mesenchymal stromal cells derived exosome-rich fractionated secretome confers hepatoprotective effect in liver injury

Apeksha Damania, Deepika Jaiman, Arun Kumar Teotia, and Ashok Kumar*

Department of Biological Sciences and Bioengineering, Indian Institute of Technology Kanpur, Kanpur-208016, UP, INDIA

Supplementary Information

Isolation of MSCs

Rats were sacrificed by cervical dislocation and placed in a biosafety cabinet where the whole body was soaked in 70% alcohol. An incision was made around the connection between the hind limbs and the trunk along the length of the leg and the calf. Muscle, ligaments, and tendons were carefully disassociated from the tibias and femurs using micro-dissecting scissors and surgical scalpels. The hip joint was cut through and the head of the femur separated from the acetabulum. The tibia distal was cut through until the union of the tibia and fibula. The remaining muscle was peeled back and soft tissue cut through. The remaining soft tissue toward the knee for both bones was scraped off. The knee joint was held and the tibia bent laterally until the bone separated from the knee. While still holding the knee, the femur was hyperextended until it separated from the knee. Both the tibias and femurs were placed in a 100-mm petri dish containing PBS with 1% antibiotic.

The two ends of each bone were excised just below the end of the marrow cavity. A 23-gauge needle attached to a 10 mL syringe was used to draw basal media from a 50 mL centrifuge tube and the needle inserted into the bone cavity. The marrow was slowly flushed out into a separate 50 mL centrifuge tube. This process was repeated at least twice until the bone became pale. The above process was performed on all the bones.

The suspension obtained was passed through a 70 µm cell strainer to remove debris obtained while flushing. The suspension obtained after filtration was centrifuged at 500g for 5 min. The pellet of cells obtained was re-suspended in DMEM supplemented with 20% FBS and 1% antibiotic and plated in T-75 flasks with media changes every 3-4 days.

Characterisation of MSCs

Colony forming units (CFU) formation assay:

Cells from passage 3 were seeded in a 100-mm petri dish at a density of 100 cells/dish in the presence of complete medium. The cells were maintained in culture for 10-14 days at 37 ºC in 5% CO_2. On the $10^{th}/14^{th}$ day, complete media was aspirated and the cells washed in PBS. The cells were then fixed in 4% parafolmaldehyde for 5-10 min and subsequently incubated in 0.5% crystal violet solution in 4% parafolmaldehyde for 30 min at room temperature. Post incubation, the plates were washed twice with distilled water.

Osteogenic differentiation:

Cells from passage 3 were seeded in a 6-well tissue culture plate at a density of 3 x 10^4 cells/well. The cells were cultured in osteogenic differentiation induction medium (DMEM supplemented with 10% FBS, 1 µM dexamethasone, 10 mM β-glycerophosphate, 50 µM ascorbate and 1% antibiotic-antimycotic solution) for 3 weeks. Media was changed every 2-3 days. After 3 weeks, the cells were stained with Alizarin-red after fixation in 10% neutral buffered formalin.

Adipogenic differentiation:

Cells from passage 3 were seeded in a 6-well tissue culture plate at a density of 3 x 10^4 cells/well. The cells were then cultured for 72 h in adipogenic differentiation induction medium

(DMEM supplemented with 10% FBS, 1 µM dexamethasone, 0.5 mM isobutylmethylxanthine, 60 µM indomethacin, 10 µg/mL insulin and 1% antibiotic-antimycotic solution). After 72 h, the induction medium was aspirated and the cells cultured for 48 h in adipogenic maintenance medium (DMEM supplemented with 10% FBS, 10 µg/mL insulin and 1% antibiotic-antimycotic solution). This cycle was repeated four times after which the cells were cultured for 1 week in adipogenic maintenance medium. After 4 weeks, the cells were stained with Oil-red-O after fixation in 10% neutral buffered formalin.

As a negative control for both osteogenic and adipogenic differentiation, the cells from passage 3 were cultured in complete medium.

Characterization of exosome-rich fractionated secretome

Confocal Microscopy

FITC was added to 100 µL aliquot of fractionated secretome (1:1000) and incubated with shaking overnight. The solution was ultra-centrifuged for 2 h at 4 ºC and the pellet washed twice with PBS. The pellet was then re-suspended in PBS and a drop placed on a glass slide and air dried.

Scanning electron microscopy (SEM)

The fractionated secretome was fixed in 2% paraformaldehyde (PFA) and then serially diluted using distilled water. Samples (1-5 µl) were added onto clean silicone chips and dried in a ventilation hood. Following gold coating, the samples were imaged at low beam energies (5.0-10.0 kV).

Transmission electron microscopy (TEM)

The pellet of fractionated secretome obtained upon isolation was first fixed in 2% PFA. The samples were then prepared using a contrasting and embedding protocol previously described

by Thery et.al which allows for the whole mount analysis of the extracellular vesicles present in the fractionated secretome.

Flow cytometry

Flow cytometric analysis of exosomes present in the fractionated secretome was performed using commercially available Exo-FACS kit (Exosome marker identification via FACS analysis) according to the manufacturer's protocol. The basic protocol included binding the exosomes on 4 µm latex FACS-beads provided in the kit. The exosome-bead complexes were incubated with primary antibody for 2 h in the dark followed by 1 h incubation with secondary antibody conjugated with Alexa 488. After incubation at room temperature, beads were washed and analysed on a Partec cyflow® space, used in its standard configuration with a 15-mW 488-nm air-cooled argon laser, and standard band pass filters for Fl-1 (530/30 nm), Fl-2 (585/42 nm) and Fl-3 (625 nm). Data acquisition and analysis was performed using Flowmax software (Partec). The beads were assessed from the dot plot representation of forward (FSC) and side scatter (SSC), set to a linear scale. From this plot, single beads could be observed. Only single beads were gated for fluorescence analysis. The mean channel fluorescence value of the histogram plot was taken as a measure of intensity. A bead only control was also included and the fluorescence intensity was normalized.

Western blot analysis

Equal amounts of fractionated secretome and MSC cell lysate (10 µg) were resuspended in RIPA lysis buffer (25 mM Tris-HCL pH 7.6, 150 mM NaCl, 5 mM EDTA, 1% NP-40, 1% sodium deoxycholate and 0.1% SDS) containing SIGMAFAST™ protease inhibitor cocktail (S8830). Lysates were bath sonicated in 5 cycles (15 s ON and 30 s OFF). Samples were resuspended in 4X-SDS loading buffer, heated for 10 min at 95 ºC, resolved on a 12% SDS-PAGE gel and transferred to a nitrocellulose membrane (MDI Membrane Technologies, India).

The membranes were blocked with 3% skimmed milk in TBS-T followed by incubation with primary antibody CD-81 (Santa Cruz, TX, USA, sc-7637) and secondary antibody goat anti-mouse IgG-HRP (Santa Cruz, TX, USA, sc-2005). The same blot was stripped for 30 min in stripping buffer and reprobed with anti – actin antibody (Santa Cruz, TX, USA, sc 10731) and secondary anti-rabbit antibody (Santa Cruz, TX, USA, sc-2004). Immunoreactive bands were visualized using Immobilon Western Chemiluminescent HRP substrate (WBKLS0500) and signal was captured using ChemiDocTM MP Imaging System (Biorad).

Sandwich ELISA

Sandwich enzyme-linked immunoassay was used to determine the presence of the biomarker CD9 on the extracellular vesicles present in the fractionated secretome. Commercially available pre-coated plates for exosome capture (HansaBioMed, Tallin, Estonia) were used for exosome ELISA assay according to manufacturer's instructions. Basic protocol typically comprised incubation with the primary antibody (aCD9) diluted in sample buffer (0.5% BSA PBS) for 2 h at 4 °C. After extensive washing, the plate was incubated with HRP-conjugated Secondary Antibody. Upon addition of the substrate optical densities were recorded at 450 nm using a microplate reader.

In vitro study for recovery of viability in presence of exosome enriched fractionated secretome

Qualitative analysis using fluorescence microscopy

At regular time intervals, media was removed and cells washed with PBS. Propidium iodide (PI) stain (2 mg/mL) (a fluorescent dye that stains only dead cells) was added to the cells drop by drop and incubated for 10-15 min in the dark. After this, fluorescein diacetate (FDA) stain (5 mg/mL) was added and incubated for 5 min. Post incubation, the cells were washed with PBS and immediately imaged using a fluorescent microscope.

Effect of MSC exosome enriched fractionated secretome on ROS accumulation due to liver injury

DCFDA assay

At known time intervals, media was aspirated and 10 µM DCFDA (diluted in serum-free DMEM) added to the cells and incubated for 30 min at 37 ºC. After incubation, the cells were washed and immediately analyzed under the fluorescent microscope or lysed using saline sodium citrate (SSC) lysis buffer. Fluorescence was measured at an excitation/emission wavelength of 495/520 nm. For fluorescence microscopy, the cells were counter stained with 4',6-diamidino-2-phenylindole (DAPI).

Effect of exosome enriched fractionated secretome on liver regeneration rate and recovery of liver functions in rodent model of liver failure

Calculation of liver regeneration rate

$$Liver\ regeneration\ rate = \frac{Remnant\ liver\ weight}{Est.whole\ liver\ weight} \tag{1}$$

wherein,

$$Est.\ whole\ liver\ weight = \frac{Resected\ liver\ weight}{0.7} \tag{2}$$

since approximately 70% of the liver was resected out.
