# Supplementary figures and images for: Mesenchymal stromal cell-derived exosome-rich fractionated secretome confers a hepatoprotective effect in liver injury
Source: Stem Cell Res Ther. 2018 Feb 6;9:31. doi: 10.1186/s13287-017-0752-6 (PMC5801895; doi:10.1186/s13287-017-0752-6)

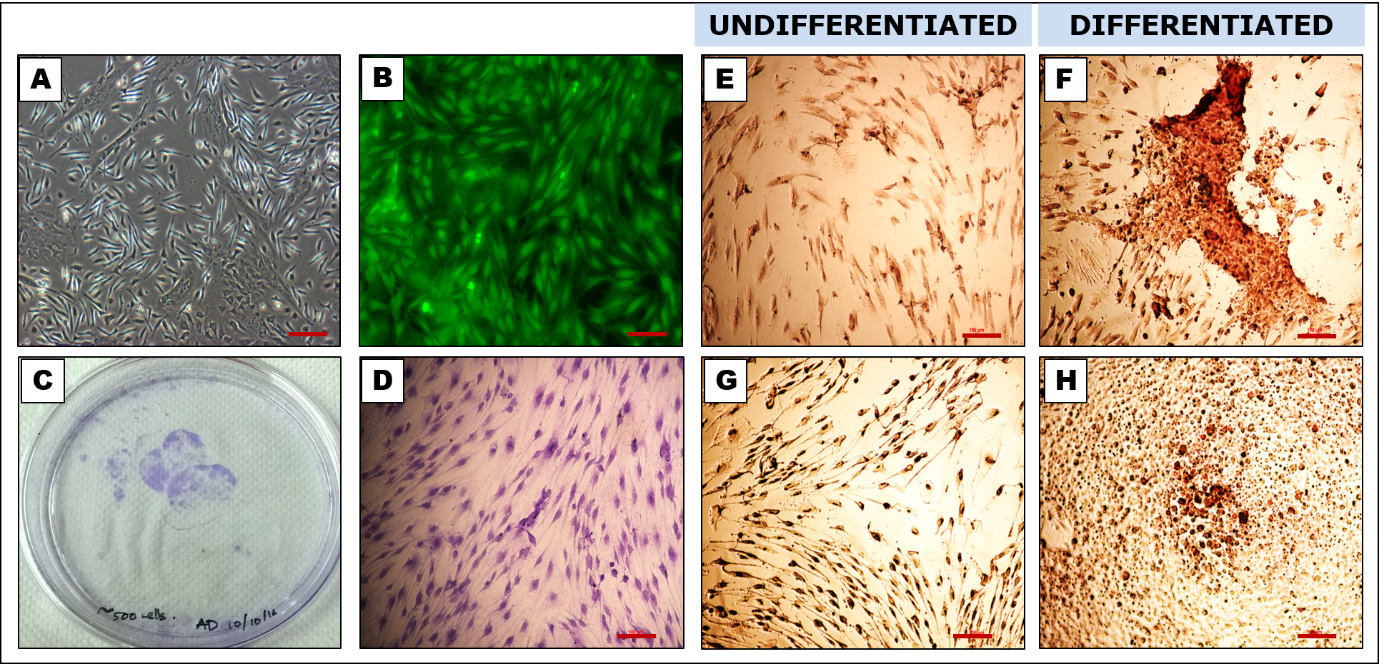

Supplement: Supplementary file 2 — showing characterization of rat bone marrow-derived mesenchymal stem cells. Phase-contrast microscopic image of cultured MSCs showing fibroblast-like spindle-shaped morphology (A), fluorescence microscopic image of MSCs stained with fluorescein diacetate (FDA) (B), digital image of crystal violet staining of colonies of MSCs formed (C), microscopic image of MSCs stained with crystal violet (D), Alizarin Red staining of undifferentiated MSCs (E) and osteogenically differentiated MSCs (F) and Oil Red O staining of undifferentiated MSCs (G) and adipogenically differentiated MSCs (H). Scale bar for all microscopic images: 100 μm (TIF 2112 kb) [file 13287_2017_752_MOESM2_ESM.tif]
